# Proteins and Signaling Pathways Response to Dry Needling Combined with Static Stretching Treatment for Chronic Myofascial Pain in a RAT Model: An Explorative Proteomic Study

**DOI:** 10.3390/ijms20030564

**Published:** 2019-01-29

**Authors:** Lihui Li, Qiangmin Huang, Marco Barbero, Lin Liu, Thitham Nguyen, Anle Xu, Lijuan Ji

**Affiliations:** 1 Department of Sport Medicine and Rehabilitation Center, Shanghai University of Sport, Shanghai 200438, China; 1610302015@student.sus.edu.cn (L.L.); 1620325008@student.sus.edu.cn (A.X.); 1620325009@student.sus.edu.cn (L.J.); 2 Rehabilitation Research Laboratory 2rLab, Department of Business Economics Health and Social Care, University of Applied Sciences and Arts of Southern Switzerland, 6928 Manno, Switzerland; marco.barbero@supsi.ch; 3 Sport and Health Science Department, Nanjing Sport Institute, Nanjing 210014, China; liulinst1989@126.com; 4 Faculty of Sport Science, Ton Duc Thang University, Ho Chi Minh City 71000, Viet Nam; nguyenthitham@tdtu.edu.vn

**Keywords:** myofascial trigger points, proteomic, chronic myofascial pain, dry needling, stretching, mass spectrometry, tandem mass tag, actinin alpha 3, calsequestrin-1, parvalbumin alpha

## Abstract

A quantitative proteomic analysis of the response to dry needling combined with static stretching treatment was performed in a rat model of active myofascial trigger points (MTrPs). 36 rats were divided into a model group (MG), a stretching group (SG) and a dry needling combined with stretching group (SDG). We performed three biological replicates to compare large-scale differential protein expression between groups by tandem mass tag (TMT) labeling technology based on nanoscale liquid chromatography mass spectrometry analysis (LC–MS/MS). Hierarchical clustering, Gene Ontology (GO), Kyoto Encyclopedia of Genes and Genomes (KEGG) enrichment and protein-protein interaction network analyses were performed for the general characterization of overall enriched proteins. For validation of the results of TMT, the candidate proteins were verified by parallel reaction monitoring (PRM) analysis. 285 differentially expressed proteins between groups were identified and quantified. Tight junction pathway played a dominant role in dry needling combined with static stretching treatment for the rat model of active MTrPs. Three candidate proteins, namely actinin alpha 3, calsequestrin-1 and parvalbumin alpha, were further validated, consistent with the results of LC–MS/MS. This is the first proteomics-based study to report the therapeutic mechanism underlying dry needling and static stretching treatment for MTrPs. Further functional verification of the potential signaling pathways and the enriched proteins is warranted.

## 1. Introduction

Pain, which is not an isolated pathological process, is often intertwined clinical problems and comorbid conditions [1,2]. Numerous studies have demonstrated that active myofascial trigger points exist in most types of pain [3,4,5,6]. Myofascial trigger points (MTrPs) are considered hyperirritable spots in taut bands of skeletal muscles, which are composed of numerous “contraction knots” in myofibers [7,8,9]. The existence of active MTrPs causes a series of clinical pain-related complaints owing to the shortening of the muscle fiber and pressure on nearby nerves or veins due to these contraction knots [10]. Therefore, the inactivation or elimination of MTrPs by various interventions may relieve chronic myofascial pain.

Dry needling is a commonly applied intervention for MTrPs [11,12,13]; clinically, static stretching is usually performed after dry needling treatment [14,15,16]. More specifically, the dry needling technique focuses on accurate punctures into the MTrPs, while stretching targets the muscles around the MTrPs. According to Simons’ integrated hypothesis, in the development of MTrPs local tissue environment changed [17] and several proinflammatory mediators for instance cytokines, calcitonin gene-related peptide, substance P, bradykinin, 5-hydroxytryptamin excessively released [18,19,20]. The peripheral pain receptors in muscle tissue are dynamic structures and if the local tissue environment altered, the conformation of the structures would be changed [17]. Our previous study investigated the changes in acetylcholine (ACh), acetylcholine esterase (AChE) and acetylcholine receptor (AChR) levels after dry needling at exact MTrPs site in a rat model. The results showed that ACh and AChR levels significantly decreased, whereas AChE significantly increased after dry needling treatment. [21]. A large number of studies have confirmed the beneficial effects of skeletal muscle stretching on muscle performance, range of motion and even neurological responses [13,14,15,16,22,23]. Recently, a study from Austria showed that one minute of static stretching increases H-reflex excitability and decreases muscle spindle sensitivity [16]. Further, our group found that H-reflex pathways were involved in the pathophysiological mechanism of MTrPs. However, such approaches did not systemically elucidate the underlying comprehensive mechanism involved in dry needling combined with static stretching for inactivation of the active MTrPs.

MS-based proteomic analyses are useful for large-scale protein studies [24] aimed at the elucidation of therapeutic mechanisms in MTrPs. However, to date, there are no studies investigating the proteomic responses to dry needling combined with static stretching for MTrPs at the protein level. Accordingly, this study was designed to investigate large-scale differential protein expression after dry needling combined with static stretching of the relevant muscles as MTrPs treatment.

## 2. Results

### 2.1. Body Weight and the Nociception Value

**Figure 1:**
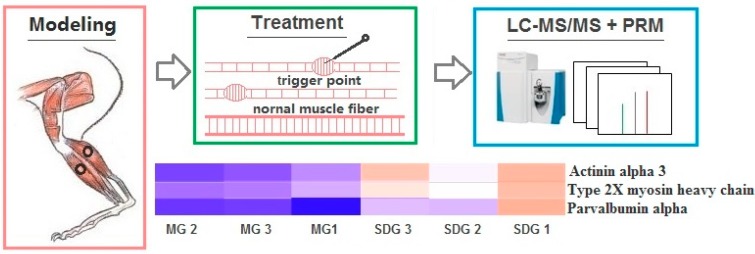
The schematic outline of the experimental workflow.

**Figure 2:**
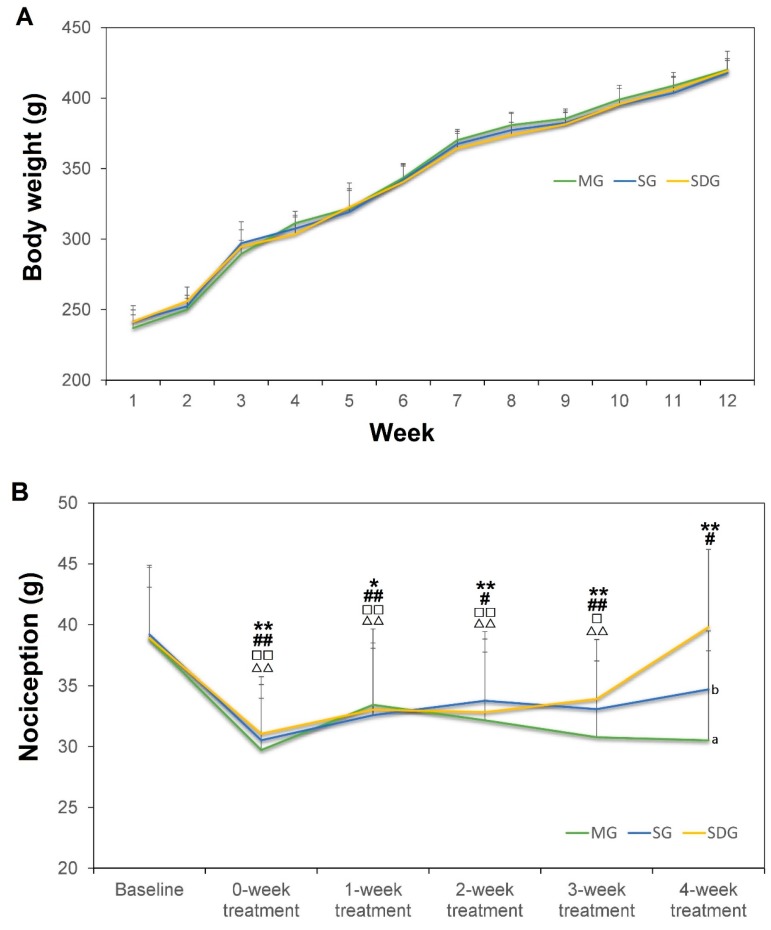
The body weight during the modeling period (**A**) and the nociception value during the treatment period (**B**), *n* = 12 for each group. Data are shown as mean ± SD. Compared to Baseline in MG group: ** *p* < 0.01, * *p* < 0.05; SG group: ^##^ *p* < 0.01, ^#^ *p* < 0.05; SDG group: ^□□^ *p* < 0.01, ^□^ *p* < 0.05. Compared to 4-week treatment in SDG group: ^∆∆^ *p* < 0.01, ^∆^ *p* < 0.05. Compared to SDG group at the 4-week treatment: ^a^ *p* < 0.01, ^b^ *p* < 0.05 by Repeated Measures ANOVA with post hoc tests.

The schematic outline of the experimental workflow is showed in Figure 1. The body weight of all rats in the three groups significantly increased weekly over the modeling period (*p* < 0.05) except from the 8th to the 9th week in MG group, the 10th to the 11th week in SG group and the 3rd to the 4th week in SDG group (*p* > 0.05). There was no statistical difference between groups (Figure 2A).

As showed in Figure 2B, the nociception of the left hind paw was significantly decreased at 0-week treatment (post-modeling/before treatment), 1-week treatment, 2-week treatment 3-week treatment compared to baseline (pre-modeling) in the three groups (*p* < 0.05). In the last week (4-week treatment), the nociception value was significantly decreased only in MG (*p* < 0.01) and SG group (*p* < 0.05) compared to baseline; there was no statistical difference between 4-week treatment and baseline in SDG; however, at the 4-week treatment the nociception value in SDG group was significantly higher than both in MG group (*p* < 0.01) and SG group (*p* < 0.05).

### 2.2. Hierarchical Cluster

**Figure 3:**
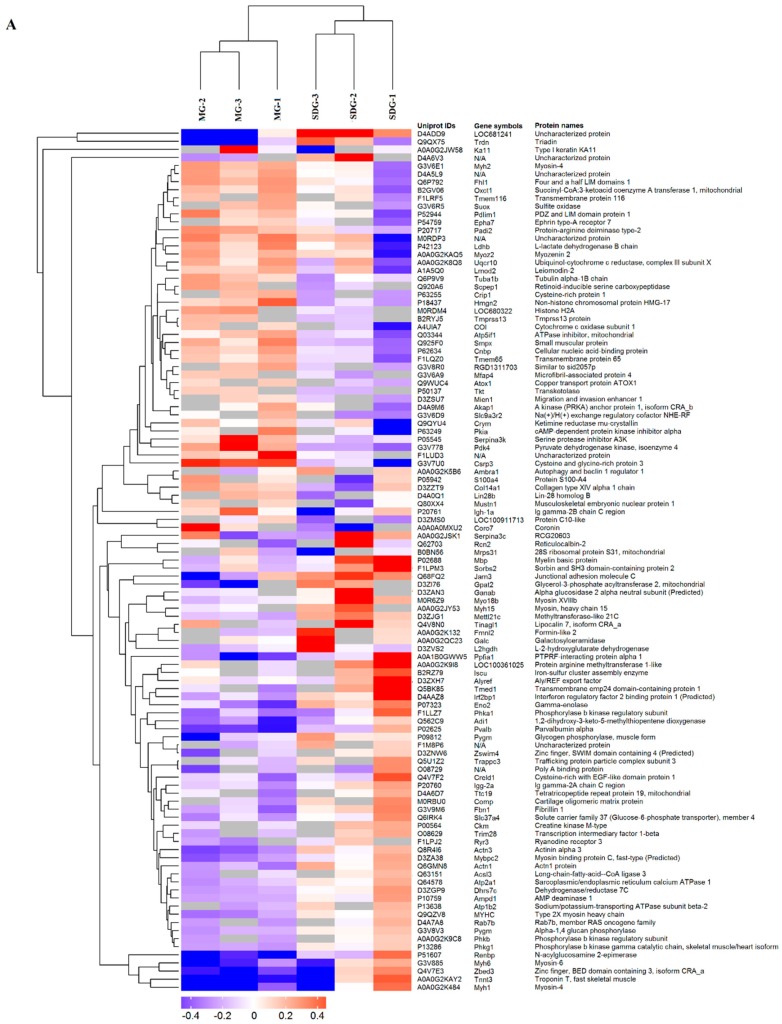

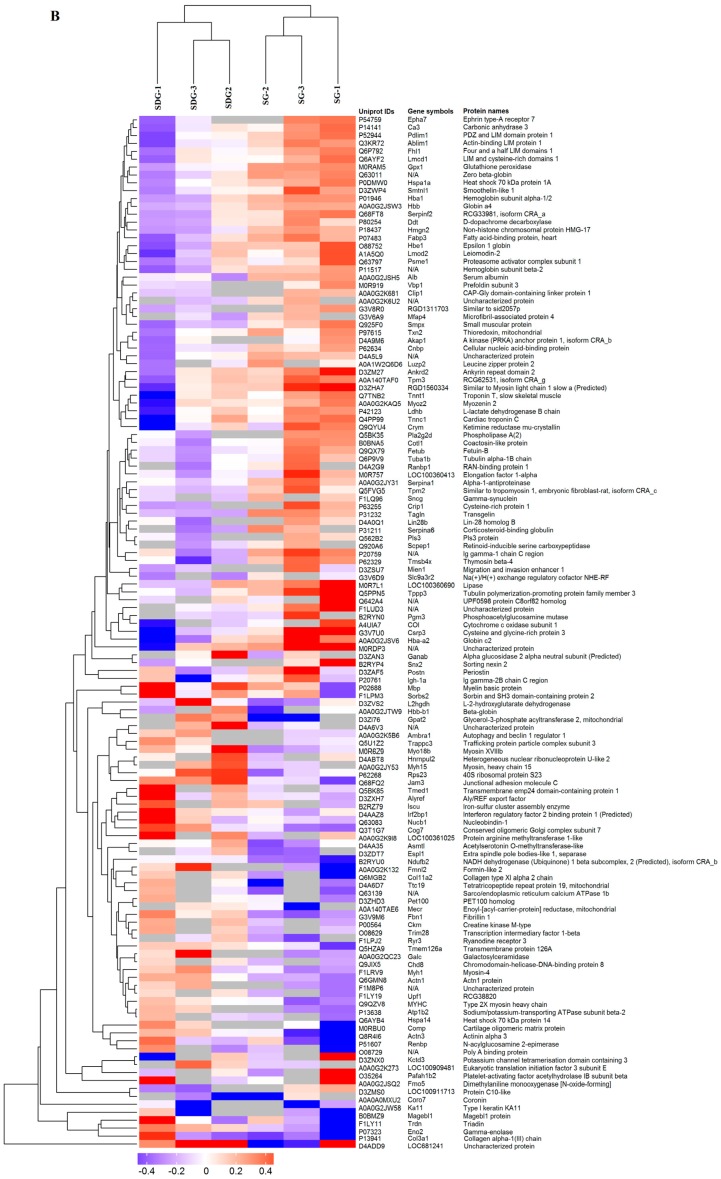

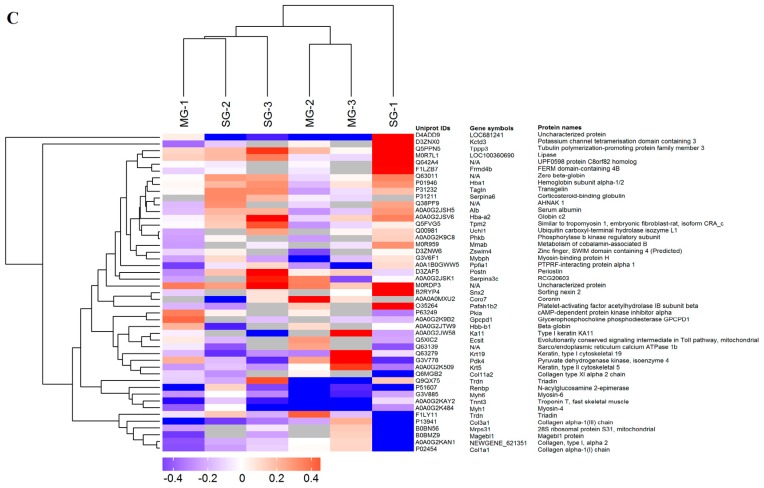
Hierarchical clustering of differently expressed proteins between SDG and model group (MG) (**A**), SDG and SG (**B**), SG and MG (**C**) groups, *n* = 3 samples for each group (3 data points for each bar), each consisting of tissue of 4 rats. Euclidean distance algorithm for similarity measurements and an average linkage clustering algorithm for clustering were selected. A heatmap was created as a visual aid, in addition to the use of a dendrogram. Each column represents one biological replicate. Each row represents one significant protein. The relative expression level was indicated using the intensity of each color. Red, blue, and gray colors indicate a relative increase, decrease, or no quantitative information in protein content for a particular protein, respectively (row).

A total of 285 differentially expressed proteins between groups were identified and quantified (Supplementary Materials). Of these, 107 proteins (Up-regulated 47; Down-regulated 60) were found between the SDG group and MG group (Figure 3A); 131 proteins (Up-regulated 76; Down-regulated 55) were found between the SG group and SDG (Figure 3B); 47 proteins (Up-regulated 30; Down-regulated 17) were found between the SG group and MG (Figure 3C). The heatmap of the hierarchical cluster analysis showed that these proteins were well distinguished, which provided improved visualization of the overall protein change (Figure 3).

### 2.3. GO and KEGG Pathway Enrichment

**Figure 4:**
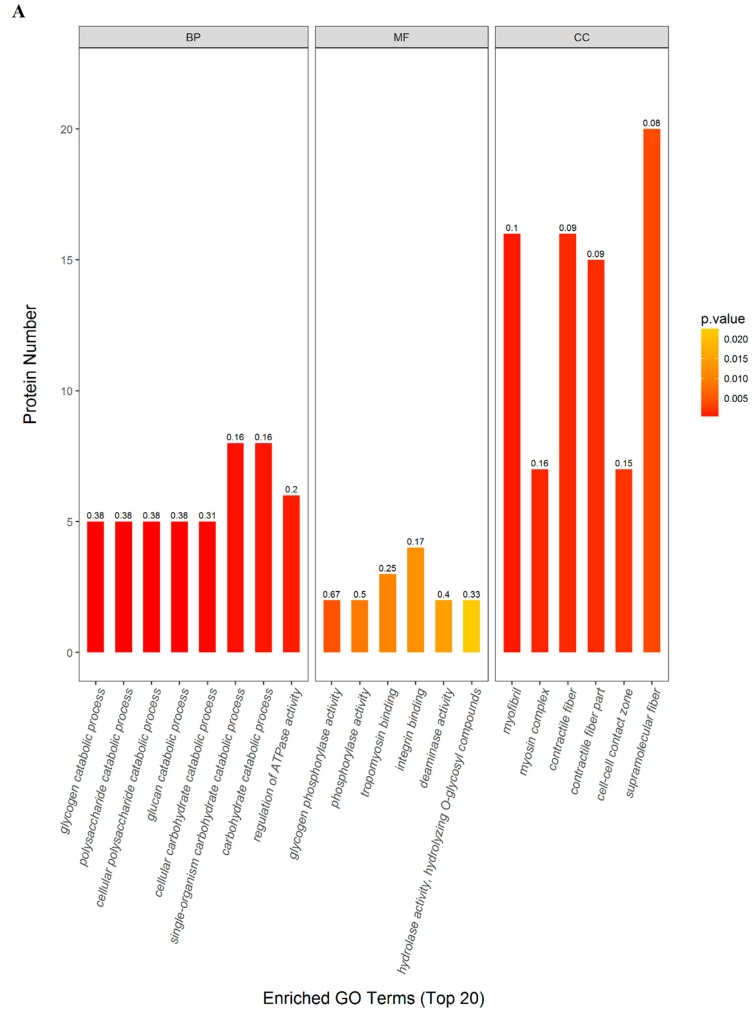

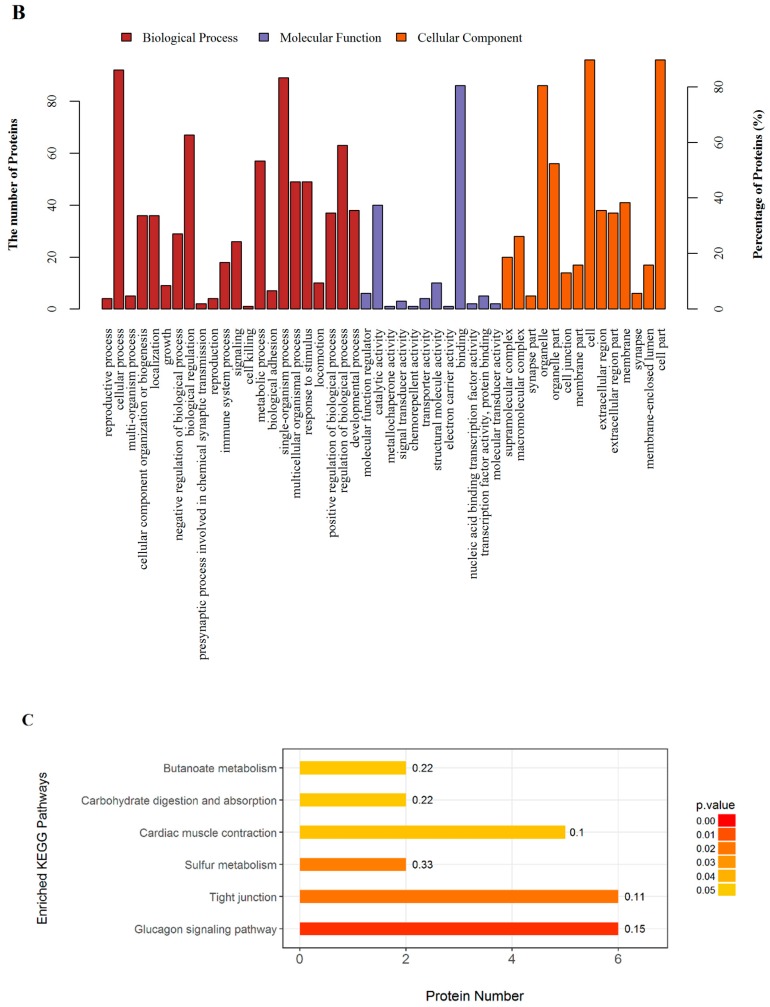
The enriched GO terms and KEGG pathways of significantly expressed proteins between SDG and MG groups, *n* = 3 samples for each group (3 data points for each bar), each consisting of tissue of 4 rats. (**A**) Top 20 enriched GO terms. The horizontal axis in the graph represents the three categories (BP: biological processes; MF: molecular functions; CC: cellular components). The vertical axes represent the number of significant proteins. The color gradient from orange to red represents the *p* value; the closer the color to red, the lower the *p* value and the higher the significance level corresponding to the enrichment. The numbers above the bar charts represent the richFactor (richFactor≤1), which is the ratio of the number of proteins that were annotated in this category. (**B**) Overall enriched GO terms. The horizontal axis with different colors of the bar charts represents the significance of enrichment of the three categories (based on 107 proteins significantly expressed proteins). The vertical axes represent the number and percentage of significant proteins. (**C**) The significantly enriched KEGG pathways. The horizontal axis in the graph represents the enriched KEGG pathways; the vertical axis represents the number of significant proteins. The colors of the bar charts represent the significant of enrichment on three categories. The color gradient from orange to red represents the *p* value; the closer to red color, the lower the *p* value and the higher the significance level corresponding to the enrichment. The numbers above the bar charts indicate the richFactor (richFactor ≤ 1), which is the ratio of significant proteins compared to the overall proteins in each category.

GO and KEGG pathway enrichment analysis were performed between SDG and MG groups. The most enriched GO terms of biological processes (BP), molecular functions (MF) and cellular components (CC) were annotated as a single-organism carbohydrate catabolic process (8 proteins, richFactor = 0.16), integrin binding (4 proteins, richFactor = 0.17) and supramolecular fibers (20 proteins, richFactor = 0.08), respectively (Figure 4A–B, Table 1).

The results of KEGG pathway analysis showed that the significant enrichment pathways included those for tight junctions (6 proteins, richFactor = 0.11), the glucagon signaling pathway (6 proteins, richFactor = 0.15), cardiac muscle contraction (5 proteins, richFactor = 0.10), sulfur metabolism (2 proteins, richFactor = 0.33), carbohydrate digestion and absorption (2 proteins, richFactor = 0.22), and butanoate metabolism (2 proteins, richFactor = 0.22) (Figure 4C, Table 1).

### 2.4. PPI Analysis

**Figure 5:**
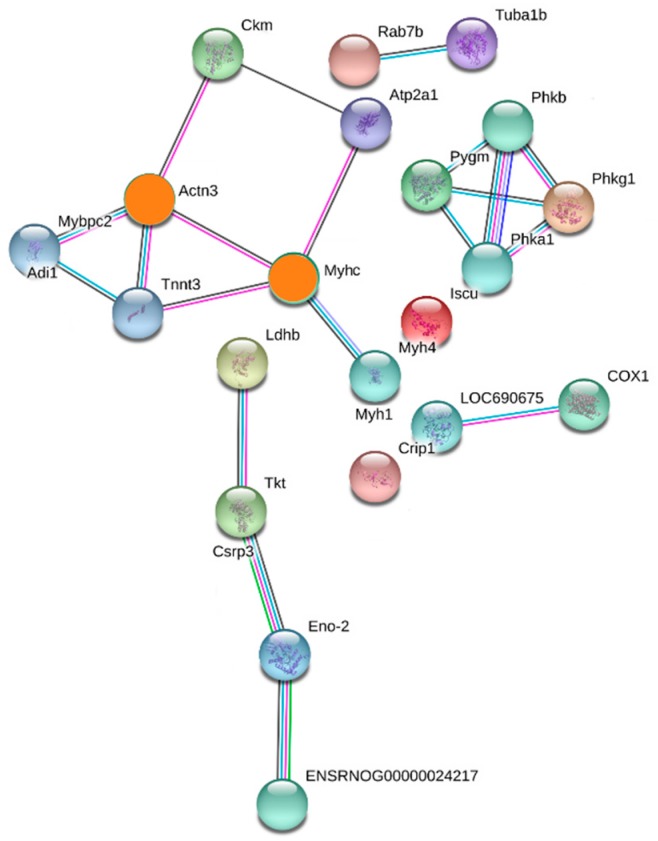
Proteins in the PPI network between SDG and MG groups. The most enriched two node proteins (marked with orange color) with high connectivity degree interacting with four significantly expressed proteins.

In the PPI network, the most enriched two node proteins with high connectivity degree between SDG and MG groups were shown as orange nodes (Figure 5): actinin alpha 3 (UniProtKB-Q8R4I6) and type 2X myosin heavy chain (UniProtKB-Q9QZV8), interacting with four significantly expressed proteins, exhibited the highest degree of connectivity (Table 2).

### 2.5. PRM Validation of TMT-Based Results

**Figure 6:**
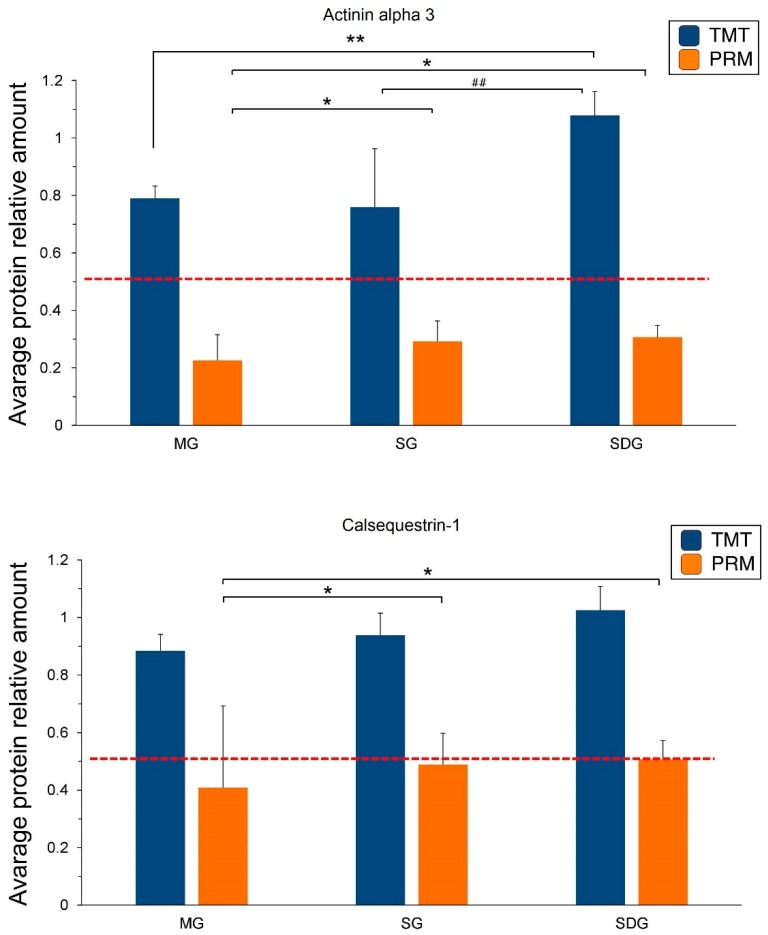

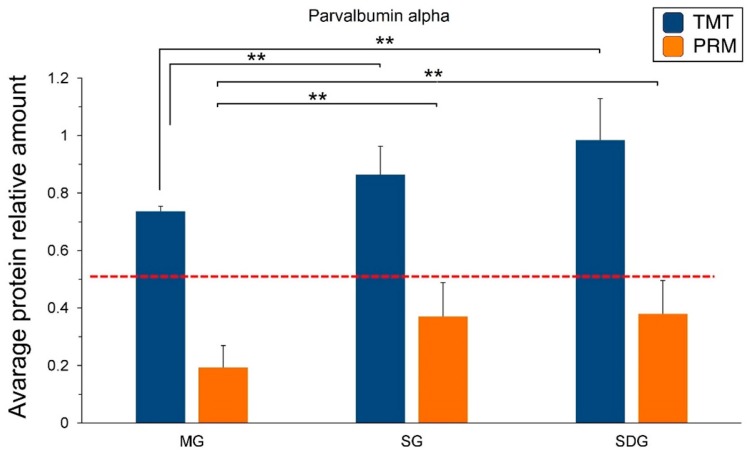
PRM and TMT protein expression quantities of the three candidate proteins, *n* = 3 samples for each group (3 data points for each bar), each consisting of tissue of 4 rats. Abbreviations: TMT, tandem mass tag; PRM, parallel reaction monitoring. Data are shown as mean ± SD, compared to MG: * *p* < 0.05, ** *p* < 0.01; compared to SG: ^##^ *p* < 0.01 by multiple t-test.

Three target proteins namely actinin alpha 3, calsequestrin-1, and parvalbumin alpha were selected for verification using LC-PRM/MS quantitative analysis. All target peptides of the three proteins have significant quantitative information in each sample. After normalized, the results of the relative quantitative expression showed that the three candidate proteins exhibited similar trends after treatment to those observed in the TMT results, which supports the plausibility and reliability of the proteomics data (Figure 6). On comparing MG to SDG, the fold change of actinin alpha 3 and parvalbumin alpha were found to be significant according to both TMT and PRM analysis. Compared to MG to SG, the change of parvalbumin alpha was significant in both the TMT and PRM analyses; while the change in calsequestrin-1 was significant only according to the PRM analysis (Figure 6).

## 3. Discussion

Previous proteomics-based studies addressing chronic pain have primarily focused on dorsal root ganglia and spinal cord tissue in animal models of neuropathic pain [25]. In the present study, rat skeletal muscle of MTrP was chosen as an “experimental model” as it may be more representative of the therapeutic mechanism underlying MTrPs. In humans, only a small number of studies have utilized proteomics to explore pain mechanisms; most of these investigations have relied on the 2D-PAGE approach coupled with mass spectrometry (MS) [25]. TMT label-based nanoscale liquid chromatography tandem mass spectrometry analysis (LC–MS/MS), as an emerging quantification technology, overcomes the deficiencies of traditional methods when macromolecule and proteins cannot be quantified [26].

Our previous study compared the healthy muscle to MTrPs muscle using proteomics technology and a total of 2635 proteins were identified [27]. This is the first study using TMT labeling proteomic technology on dry needling combined with static stretching treatment of MTrPs. In the present work, 285 with significantly differential expression were identified in three biological replicates, and hierarchical cluster analysis showed that these proteins were well distinguished. These results indicated that the overall proteins were screened with reasonable accuracy. Additionally, we verified three differentially expressed candidate proteins using MS-based precise quantitative PRM analysis, and the data showed the data showed trends that paralleled those observed for TMT results. The PRM method is conceptually similar to western blotting, but independent of the specificity of the antibody, making it superior to western blotting, especially when a high-quality antibody is unavailable [28]. Hence, the results of these MS experiments are undoubtedly technically reliable.

GO and KEGG enrichment results indicated that dry needling combined with static stretching treatment significantly regulated the tight junction pathway. Recent reports indicate that inflammatory pain may be controlled via the modulation of proteins in tight junctions [29,30,31,32]. Another study suggested that changes in tight junction play a role in the pathophysiological mechanisms underlying headache [33]. Accordingly, we speculate that the tight junction pathway (as well as significantly expressed proteins such as myosin-4, tubulin alpha-1B chain, junctional adhesion molecule C, actin1 protein and myosin heavy-chain 15 in this pathway) may represent potential therapeutic targets for MTrPs. The modulating effect of dry needling combined with static stretching may block pain signals through the tight junctions and those significant related proteins. This is the first report to implicate changes in tight junctions in skeletal muscles as a possible mechanism underlying the therapeutic effect of dry needling combined with static stretching in MTrPs.

The first candidate protein, actinin alpha 3, exhibited the highest connectivity degree on comparing dry needling combined with static stretching treatment and no treatment, by PPI analysis. In the general population, actinin alpha 3 is associated with the fast muscle-fiber area, muscle mass, strength and muscle function. The Actn3 knock-out changed muscle adaptation in response to immobilization and denervation [34]. Additionally, an interesting study showed that the fiber-type-specific expression of alpha-actinin-2 and -3 in rats resembles that of humans more than closely that of mice [35]. Moreover, actinin alpha 3 plays an important role in cross linking in actin and other structural proteins to maintain an ordered myofibrillar array of the Z-disk in skeletal muscle. During the development of MTrPs, the link between myosin and actin weakens [7,17]. In our study, the expression of actinin alpha 3 significantly improved after treatment of the rat MTrPs; this finding suggests that such changes in actinin alpha 3 may be involved in the therapeutic effects of dry needling combined with static stretching.

A lack of calsequestrin-1 results in increased oxidative stress and mitochondrial damage in skeletal muscle [36]. Static light scattering experiments and crystallographic studies found that Ca^2+^ dependent polymerization capabilities of calsequestrin are enhanced, providing a dynamic “guiderail”-like scaffold for calsequestrin polymerization [36]. Bron and Dommerholt [6] hypothesized that, following muscle injury, the cell membranes and sarcoplasmic reticulum were damaged, leading to the release of large amounts of Ca^2+^ during the development of MTrPs. In this study, according to PRM analysis, calsequestrin-1 significantly increased after dry needling combined with static stretching or simple static stretching treatment alone. Although this increase was not significant according to TMT-based analysis (this outcome may be affected by many factors, and a high false-positive rate should be considered), the current findings provide novel insights into potential therapeutic strategies for MTrPs.

The calcium-binding protein, parvalbumin alpha, maintains the exchange of intracellular Ca^2+^. Activation of parvalbumin neurons in the cerebral cortex has been shown to increase pain sensitivity [37], and dorsal horn parvalbumin neurons are considered gate-keepers of touch-evoked pain that alleviate mechanical allodynia in neuropathic pain [38]. However, the role of parvalbumin alpha in muscle tissue function related to musculoskeletal pain or MTrP-related pain is not clear. Our findings demonstrated that dry needling combined with static stretching, or simply static stretching treatment alone, significantly increased the expression of parvalbumin alpha in the gastrocnemius muscle in the present MTrP model. These results identify parvalbumin alpha as another potential target for the treatment of MTrPs.

## 4. Materials and Methods

### 4.1. MTrPs Modeling

All animal experiments were conducted at the Laboratory Animal Center of Shanghai University of Sport, China. The procedure was approved by the Ethics Committee of the Shanghai University of Sport (approval code: 2017017 and approval date 07/03/2017). The experiments complied with the ARRIVE (Animal Research: Reporting of in Vivo Experiments) guidelines. A total of 36 male Sprague-Dawley rats (7-week age, 220 to 250 g, Shanghai Jiesijie Experimental Animal Co. Ltd., Shanghai, China) were housed in groups of three or five in an specific pathogen-free (SPF) animal experimental laboratory room under a 12 h light/dark cycle, and randomly divided into three groups by a block randomization method using a web-based random number generator (GraphPad software, San Diego, CA, USA) (*n* = 12 each group): a model group (MG), a stretching group (SG), and a dry needling combined with stretching group (SDG). The rat model of active MTrPs was established by blunt striking on the left gastrocnemius muscles (GM) and eccentric-based exercise for 8 weeks along with 4 weeks of recovery. The left GM of rats in all groups was marked and struck by a hand-made stick device dropped from a height of 20 cm with a kinetic energy of 2.352J once every first day of each week; then, rats were run on a treadmill (DSPT-202, Duanshi Co., Hangzhou, China) for 1.5 h at a −16° downward angle and speed of 16 m/min every second day of each week. The general health of the rats during the procedures such as body weight, condition of the limbs, and movement of all rats was monitored weekly.

To identify the active MTrP, first a contracture nodule in a taut band was palpated and marked, and an electrode needle was inserted into the marked nodule; if local twitch responses (LTRs) were found to be elicited by needling, the marked nodule was considered a possible active MTrP. Second, for confirmation, an EMG device with bipolar electrodes (Z2J-NB-NCC08, NCC Medical Co., Ltd., Shanghai, China) was used to verify the potential MTrP. The first needle was kept in situ as one electrode, and a second electrode needle was longitudinally inserted 3–5 mm away from the first as a reference electrode. If the spontaneous electrical activity (SEAs) were recorded within 60 s, it was considered a successfully modeled MTrP [21].

The nociceptive mechanical threshold from left hind paw was measured before and after modeling by the electronic Von Frey method ((BME-40 electronic Von Frey, Tianjin Bern technology LTD, Tianjin, China). The test tip has a diameter of 0.4 mm^2^ and an area of 0.12 mm^2^ and the force range is 0.1–50 g FORCE. The test tip was placed on the nodule site that can be palpated at the model, and the investigator applied the tip in the muscle region with a gradual increase in pressure. The intensity value was recorded in grams (g) when the rat produces the paw flexion reflex action.

### 4.2. Static Stretching and Dry Needling Treatment

After the MTrPs model had been successfully generated, the corresponding dry needling and/or static stretching treatment was adopted, respectively, in the SG and SDG groups once a week, for 4 weeks. In the SG group, the rats were gently placed in a supine position in the palm of one researcher’s hand (to ensure that the rat’s body was straight and not bent or curled in the palm). Then, the left hip joint and knee joint were slowly stretched to 180°, and the ankle joint extended to 90° by another researcher. The static stretching position was maintained for 1 min, followed by rest for 1 min; this sequence was repeated three times. This stretching treatment was performed by two skilled researchers each time. In the SDG group, the rats were anesthetized and then fixed on a board. An acupuncture needle (Ф 0.3 mm) was rapidly inserted and withdrawn precisely at each single MTrP on the GM to obtain six LTRs [39]. After dry needling, static stretching, as in the SG group, was performed immediately. The nociceptive mechanical threshold from left hind paw was measured every week during the treatment period.

### 4.3. Proteomics Analysis

#### 4.3.1. Tandem Mass Tag (TMT) Sample Preparation

After the treatment was terminated on the 5th day, all rats in each group were deeply anesthetized (with sodium pentobarbital, 60 mg/kg i.p.) and the left GM tissues were quickly removed, rapidly frozen in liquid nitrogen, and maintained for 5 min. Four individual samples from the same group were pooled to constitute a pool. A total of nine pooled samples were prepared (3 data points for each group), each consisting of tissue of 4 rats.

The SDT lysis buffer (150 mM Tris-HCl, 4% SDS, 100 mM DTT, pH 8.0) was added to the sample and transferred to 2 mL tube. The lysate was homogenized twice for 60 s using an MP homogenizer (24 × 2, 6.0 M/S). The homogenate was sonicated and then boiled for 15 min. After centrifuging at 14000× *g* for 40 min, the supernatant was filtered with 0.22 µm filters. The filtrate was quantified with the BCA Protein Assay Kit (Bio-Rad Inc., Hercules, USA). Protein samples of 20 µg (each sample) were mixed with 5X loading buffer and the proteins were separated on 12.5% SDS-PAGE gel for 90 min with a constant current of 14 mA after boiling for 5 min.

Filter-aided sample preparation (FASP) and digestion [40]: firstly, 200 μg of proteins (each sample) were added to a sample of 30 μL of SDT buffer and then the detergent, DTT and other low-molecular-weight components were removed using UA buffer (8 M Urea, 150 mM Tris-HCl pH 8.0) by repeated ultrafiltration (Microcon units, 10 kD). Then 100 μL iodoacetamide (IAA) was added to block the reduced cysteine residues. The samples were then incubated for 30 min in the darkness. Then, the filters were washed in triplicate with 100 μL uric acid buffer and washed twice in100 μL of 100 mM tetraethyl-ammonium bromide (TEAB) buffer. Finally, 4 μg of trypsin in 40 μL of TEAB buffer was used to digest the protein suspensions with overnight at 37 °C, and the resulting peptides were collected as a filtrate. Concentration of the peptides can be estimated by UV spectrometer assuming that 0.1% solution of vertebrate proteins has at 280 nm an extinction of 1.1 absorbance units.

Following sample digestion and immediately before use, the TMT labeled reagents were equilibrated to room temperature, and 3-plex TMT reagent was applied to label 100 μg of the peptide mixture of each sample, according to the instructions of the manufacturer (Thermo Fisher Scientific Inc., Waltham, USA). The peptides were labeled with TMT-127, TMT-130, and TMT-131 for the MG, SG, and SDG groups for three biological replicates. The incubation reaction was performed for 1 h, and 8 μL of 5% hydroxylamine was added to the sample; then, the incubation was allowed to proceed for 15 min.

TMT-labeled samples were fractionated into 10 fractions using a Pierce high-pH reversed-phase fractionation kit (Thermo Fisher Scientific, Waltham, MA, USA) by an increasing acetonitrile step-gradient elution. Before nanoscale liquid chromatography spectrometry analysis (LC–MS/MS) analysis, the fractions were dissolved in 0.1% formic acid according to the instructions of the manufacturer (Thermo Fisher Scientific, Waltham, MA, USA).

#### 4.3.2. LC-MS/MS Analysis

The fractionated peptide mixture was placed onto a reverse phase trap column (Home-made column, 100 μm × 20 mm, 5 μm-C18) coupled with the C18-reversed phase analytical column (Home-made tip-column, 75 μm × 200 mm, 3 μm-C18) in a 0.1% formic acid buffer. Then, the mixture was separated with a linear gradient of the other buffer (0.1% formic acid and 84% acetonitrile). The flow rate was controlled at 300 nl/min using IntelliFlow technology.

A quadrupole Q-Exactive mass spectrometer coupled to an Easy nLC (Thermo Fisher Scientific, Waltham, MA, USA) was used to perform LC-MS/MS analysis, and the duration was set to 60 min. The instrument was run under the peptide recognition enablement mode. A data-dependent top-20 method was used to acquire the MS data. The most abundant precursor ions for higher energy collisional dissociation (HCD) fragmentation were dynamically selected from the survey scan at a speed of 300 to 1800 *m*/*z*. The dynamic exclusion duration was set to 40 s, and the maximum injection time was 10 ms, and the automatic gain control (AGC) target was 3 × 10^6^. The HCD spectrum resolution was set to 17,500 at 200 *m*/*z* (isolation width 2 *m*/*z*, TMT 3-plex) and the resolution for survey scans was 70,000 at 200 *m*/*z*. The under-fill ratio was defined as 0.1% and the normalized collision energy was set to 30 eV.

The MASCOT engine (version 2.2, Matrix Science, London, UK) with Proteome Discoverer 1.4 was used to search MS/MS spectra. Filtered MS/MS spectra were searched against the UniProtKB *Rattus norvegicus* decoy database, including both regular and reversed protein sequences for estimation of false-positive rates. The data were based on a false discovery rate (FDR) ≤ 1% confidence for protein identification. A threshold of proteins relative expression ratios ≥ 1.2-fold increase or ≤ 0.83-fold decrease were considered as significantly different expression with a statistic confidence of *p*-value < 0.05 between two groups.

#### 4.3.3. Bioinformatics Analysis

A bioinformatics analysis between the SDG group and the MG group was performed. Cluster3.0 and Java TreeView software was used to study relative protein expression data by performing a hierarchical clustering analysis. The protein sequences of differentially expressed proteins were retrieved in batches from the UniProtKB database in FASTA format. The NCBI BLAST+ software was used to identify homolog sequences and transferred the functional annotation to the studied sequences (http://www.blast2go.com/). Top-20 blast hits (E-value < 1 × 10^−3^) for each query sequence were retrieved and loaded into Blast2GO (Version 3.3.5) for gene ontology (GO) mapping and annotation. The protein sequences of differentially altered proteins between the two groups were blasted against the online kyoto encyclopedia of genes and genomes (KEGG) database (http://www.genome.jp/kegg/). KEGG_12_ was subsequently used for mapping the pathways. Only functional categories and pathways with *p*-values ≤ 0.05 were considered significant. The protein–protein interaction (PPI) information of the studied proteins was retrieved from IntAct molecular interaction database by their gene symbols. The results were downloaded in the XGMML format and imported into Cytoscape software package (version 3.2.1) to visualize and further analyze functional protein-protein interaction networks. Furthermore, to evaluate the importance of the protein in the PPI network, the degree of each protein was calculated.

### 4.4. Parallel Reaction Monitoring (PRM) Validation

To verify the results of TMT analysis coupled with LC–MS/MS, PRM was performed among all proteins of differential abundance under each condition. Three proteins of interest, namely actinin alpha 3, calsequestrin-1, and parvalbumin alpha, were selected for analysis. The remaining sample of TMT proteome was used for direct enzymatic hydrolysis. 200 µg protein was taken from each sample of each group separately, and 10 fmol heavy isotope-labeled peptide DSPSAPVNVT**V**R was incorporated as internal standard. DTT was added to a final concentration of 10 mM, and then heated on the boiling water bath for 15 min following cooling it to room temperature. 200 μL UA buffer (8 M Urea, 150 mM Tris-HCl, Ph 8.0) was then mixed in it after centrifuging at 14000× *g* for 30 min. 100 μL IAA (50 mM IAA in UA) was added with 600 rpm shaking for 1 min. 100 μL NH_4_HCO_3_ buffer (50 mM) was added after centrifuging at 14000× *g* for 20 min twice. Then 40 μL NH_4_HCO_3_ buffer (4 μg Trypsin) was added after shaking at 600 rpm for 1 min. 40 μL NH_4_HCO_3_ buffer (50 mM) was added after centrifuging at 14000× *g* for 30 min. The filtrate was collected. The digested peptides were desalted and lyophilized, then reconstituted with 0.1% formic acid, and the peptide concentration was determined by OD280.

Chromatographic separation was carried out using the HPLC system Easy nLC. Buffer A is 0.1% formic acid aqueous solution. Buffer B is acetonitrile with 0.1% formic acid aqueous solution (acetonitrile 84%). Samples were added to the trap column and then gradient-separated using a Thermo Fisher Scientific EASY column at speed of 300 nL/min. PRM mass spectrometric analysis was performed using a Q-Exactive HF instrument (Thermo Fisher Scientific, Waltham, MA, USA). The full scan at the first MS was performed at a resolution of 60,000 (@ 200 *m*/*z*), with a scan mass range of 300 to 1800 *m*/*z* for 60 min. AGC target: 3 × 10^6^; Maximum IT: 200 ms. Twenty PRM (MS2 scans) were collected according to the inclusion list after each full MS scan. The secondary MS parameters were as follows: isolation window: 1.6 Th; resolution 30,000 (@ 200 *m*/*z*); AGC target: 3 × 10^6^; Maximum IT: 120 ms; MS2 Activation Type: HCD; Normalized collision energy: 27. The SEQUEST HT database with Proteome Discoverer 1.4 was used to analyse MS raw files. Raw files were first converted to centroid mzXML and Filtered MS/MS spectra were searched against the UniProtKB *Rattus norvegicus* decoy database, including both regular and reversed protein sequences for estimation of false-positive rates. The search parameters are set as follows: enzyme: trypsin; missed cleavage: 0; precursor ion mass tolerance: ±10 ppm; fragment ion mass tolerance: 0.02 Da; semi-tryptic termini and two missing cleavages were allowed; fixed modification: carbamidomethylation (C). The data were based on a false discovery rate (FDR) ≤ 0.01% confidence for protein identification. The generated .msf file was used to create a library in Skyline (version 3.5.0, Proteome Discoverer 1.4, Thermo Scientific, Waltham, MA, USA) [41] and the cut-off score was set as 0.95. Peptide probabilities were calculated by the percolator algorithm in Proteome Discover and false discovery rate was set to 0.01. We manually checked and corrected peak picking according to MS/MS spectra, retention time, the transitions, and mass accuracy. After normalizing the quantitative information by the heavy isotope-labeled peptide, a relative quantitative analysis was performed on the target peptide and the target protein.

### 4.5. Statistical Analysis

Proteomics analysis was performed in triplicate (3 data points for each group, each consisting of tissue of 4 rats), similar results were obtained on at least three separate studies, and the mean value was calculated. Statistical analysis was performed with SPSS Statistics version 21.0. The data were presented as the mean ± SD. GO enrichment on three categories (biological process, molecular function, and cellular component) and KEGG pathway enrichment were analyzed by the Fisher’ exact test based on the entire quantified protein annotations as the background dataset. To adjust derived *p*-values, Benjamini-Hochberg correction was further applied for multiple testing. Only functional categories and pathways with *p*-values < 0.05 were considered as significant. Multiple t-test or Repeated Measures ANOVA with post hoc tests was performed to analyze the quantitative variables and a *p*-value < 0.05 was considered to be statistically significant.

## 5. Conclusions

In conclusion, this is the first study to utilize a TMT-based proteomic approach for the identification of large-scale differential protein expression after dry needling combined with static stretching treatment for MTrPs in a rat model. The tight junction pathway was considered to represent a potential mechanism involved in the improvement of MTrP in response to dry needling combined with static stretching treatment. Three proteins (actinin alpha 3, calsequestrin-1, and parvalbumin alpha) may provide valuable clues towards the elucidation of therapeutic mechanisms underlying dry needling of MTrP sites and static stretching. Future studies should aim towards the functional validation of the relevant proteins and signaling pathways.

## Figures and Tables

**Table 1:** Distribution of proteins and signaling pathways response to dry needling combined with static stretching treatment based on GO and KEGG analysis.

**Terms**	**Count**	**P value**	**FDR (%)**	**richFactor**	**Protein Names (*Gene Names*)**
**GO (gene ontology)**					
(BP) Single-organism carbohydrate catabolic process	8	0.0006	0.2982	0.1633	Phosphorylase b kinase gamma catalytic chain, skeletal muscle/heart isoform (*Phkg1*); Phosphorylase b kinase regulatory subunit (*Phka1*); Phosphorylase b kinase regulatory subunit (*Phkb*); Glycogen phosphorylase, muscle form (*Pygm*); L-lactate dehydrogenase B chain (*Ldhb*); Actinin alpha 3 (*Actn3*); Alpha-1,4 glucan phosphorylase (*Pygm*); Gamma-enolase (*Eno2*)
(MF) Integrin binding	4	0.0125	0.4795	0.1739	Actinin alpha 3 (*Actn3*); Fibrillin 1 (*Fbn1*); Actn1 protein (*Actn1*); Junctional adhesion molecule C (*Jam3*)
(CC) Supramolecular fiber	20	0.0040	0.4795	0.0755	Type I keratin KA11 (*Ka11*); Actinin alpha 3 (*Actn3*); Troponin T, fast skeletal muscle (*Tnnt3*); Myosin-4 (*Myh2*); Fibrillin 1 (*Fbn1*); Small muscular protein (*Smpx*); Actn1 protein (*Actn1*); Tubulin alpha-1B chain (*Tuba1b*); Myosin, heavy chain 15 (*Myh15*); Sarcoplasmic/endoplasmic reticulum calcium ATPase 1 (*Atp2a1*); Type 2X myosin heavy chain (*Myhc*); Myosin-4 (*Myh1*); Myosin-6 (*Myh6*); Cysteine and glycine-rich protein 3 (*Csrp3*); Uncharacterized protein; Leiomodin-2 (*Lmod2*); Myosin binding protein C, fast-type (*Mybpc2*); Microfibril-associated protein 4 (*Mfap4*); Myozenin 2 (*Myoz2*); Ryanodine receptor 3 (*Ryr3*)
**Terms**	**Count**	**P value**	**FDR (%)**	**richFactor**	**Protein Names (*Gene Names*)**
**KEGG (kyoto encyclopedia of genes and genomes) pathways**					
Tight junction	6	0.0189	0.7500	0.1132	Myosin-4 (*Myh2*); Tubulin alpha-1B chain (*Tuba1b*); Myosin-4 (*Myh1*); Junctional adhesion molecule C (*Jam3*); Actn1 protein (*Actn1*); Myosin, heavy chain 15 (*Myh15*)
Glucagon signaling pathway	6	0.0042	0.4642	0.1538	L-lactate dehydrogenase B chain (*Ldhb*); Protein arginine methyltransferase 1-like (*LOC100361025*); Glycogen phosphorylase, muscle form (*Pygm*); Phosphorylase b kinase regulatory subunit (*Phka1*); Alpha-1,4 glucan phosphorylase (*Pygm*); Phosphorylase b kinase gamma catalytic chain, skeletal muscle/heart isoform (*Phkg1*)
Sulfur metabolism	2	0.0220	0.7501	0.3333	Uncharacterized protein; Sulfite oxidase (*Suox*)
Cardiac muscle contraction	5	0.0464	0.7501	0.1020	Cytochrome c oxidase subunit 1 (*COI*); Ubiquinol-cytochrome c reductase, complex III subunit X (*Uqcr10*); Myosin-6 (*Myh6*); Sodium/potassium-transporting ATPase subunit beta-2 (*Atp1b2*); Sarcoplasmic/endoplasmic reticulum calcium ATPase 1 (*Atp2a1*)
Carbohydratedigestion and absorption	2	0.0488	0.7501	0.2222	Sodium/potassium-transporting ATPase subunit beta-2 (*Atp1b2*); Solute carrier family 37 (Glucose-6-phosphate transporter), member 4 (*Slc37a4*)
Butanoate metabolism	2	0.0488	0.7501	0.2222	Succinyl-CoA:3-ketoacid coenzyme A transferase 1, mitochondrial (*Oxct1*); L-2-hydroxyglutarate dehydrogenase (*L2hgdh*)

Abbreviations: BP, biological processes; MF, molecular functions; CC, cellular components; FDR, false discovery rate. GO and KEGG pathway enrichment were analyzed by the Fisher’ exact test based on the entire quantified protein annotations as the background dataset. Only functional categories and pathways with *p*-values < 0.05 were considered as significant.

**Table 2:** 4-connected proteins of Actinin alpha 3 and type 2X myosin heavy chain.

Degree	Uniprot ID	Protein Name	Gene Name
Actinin alpha 3 (4-connected)	D3ZA38	Myosin binding protein C, fast-type	*Mybpc2*
	P00564	Creatine kinase M-type	*Ckm*
	Q9QZV8	Type 2X myosin heavy chain	*Myhc*
	A0A0G2KAY2	Troponin T, fast skeletal muscle	*Tnnt3*
Type 2X myosin heavy chain (4-connected)	A0A0G2KAY2	Troponin T, fast skeletal muscle	*Tnnt3*
	Q8R4I6	Actinin alpha 3	*Actn3*
	Q64578	ATPase 1	*Atp2a1*
	A0A0G2K484	Myosin-4	*Myh1*

## Supplementary Materials

Supplementary materials can be found at http://www.mdpi.com/1422-0067/20/3/564/s1.
